# Multi-scale spatial transcriptomic atlas of the whole mouse spinal cord

**DOI:** 10.64898/2026.10.24.761397

**Published:** 2026-10-28

**Authors:** Guang-Wei Zhang, Jing Li, Junxiang J. Huang, Huizhong Whit Tao, Ke K. Zhang, Li I. Zhang

## Abstract

The spinal cord coordinates sensory, motor and autonomic functions through diverse neuronal and non-neuronal populations, yet how molecularly defined cell types are organized across its full extent remains incompletely understood. Here we use Stereo-seq to generate a genome-wide, single-cell-resolution spatial atlas of the whole adult mouse spinal cord. Profiling 322,827 cells across 81 anatomically registered transverse sections spanning cervical, thoracic, lumbar and sacral levels, we resolve 12 major cell classes and 94 transcriptomic subclusters. Major cell classes occupy characteristic anatomical territories conserved along the rostrocaudal axis, whereas individual subclusters exhibit reproducible laminar, mediolateral and segment-associated distributions. Transcriptome-wide analysis identifies coordinated spatial gene-expression modules that delineate anatomical domains independently of predefined cell-type labels. Notably, oligodendrocyte subtypes exhibit a distinct and reproducible laminar organization within spinal cord white matter across spinal levels, revealing finer structural subdivisions beyond conventional anatomical descriptions. Integrating cell-type identities with local spatial organization further defines hierarchical domains that recapitulate canonical anatomy while resolving finer subdivisions within established regions. Together, these findings define a multi-scale cellular architecture linking transcriptomic diversity, spatial gene-expression programs and anatomical organization throughout the spinal cord.

## Full Text Availability

The license terms selected by the author(s) for this preprint version do not permit archiving in PMC. The full text is available from the preprint server.

